# Analysis of Marine-Pilot Biometric Data Recordings during Port-Approach Using a Full-Mission Simulator

**DOI:** 10.3390/s22072701

**Published:** 2022-03-31

**Authors:** Dejan Žagar, Matija Svetina, Tanja Brcko, Marko Perkovič, Franc Dimc, Andrej Košir

**Affiliations:** 1 Faculty of Maritime Studies and Transport, University of Ljubljana, 6320 Portorož, Slovenia; tanja.brcko@fpp.uni-lj.si (T.B.); marko.perkovic@fpp.uni-lj.si (M.P.); franc.dimc@fpp.uni-lj.si (F.D.); 2 Faculty of Arts, University of Ljubljana, 1000 Ljubljana, Slovenia; matija.svetina@ff.uni-lj.si; 3 Faculty of Electrical Engineering, University of Ljubljana, 1000 Ljubljana, Slovenia; andrej.kosir@lucami.fe.uni-lj.si

**Keywords:** full mission nautical simulator, biometrical measurement, marine pilots, heart rate, blood-volume pulse

## Abstract

The purpose of this study is to analyse data from the marine pilots’ bio-sensor readings to determine how experience affects their biometrical response during the port approach. The experiences play a significant role in the participant’s decision-making process and correlate with the repetitions. Through the repetitions of the experimental task, the participants gain experience, which correlates with the biometrical response, e.g., heart rate, electrodermal activity, etc. After exposing the two experience-distinct groups of participants to the same simulated port-approaching task, their collected biometric data is analysed and discussed. The results show that biometrical readings of the less experienced participants typically vary compared to that of the experienced participants, who take the simulated task more seriously. The study also yields insight into the workload process, involving disturbing factors during the task.

## 1. Introduction

In this paper, the marine pilots’ biometry is measured during the port approach using a full-mission simulator. The aim is to analyse biosensor data to gain a deeper understanding of the pilots’ bio-response during the simulated task, which may include a reasoning human factor error, thus leading to a potentially dangerous situation [1].

**Figure 1:**
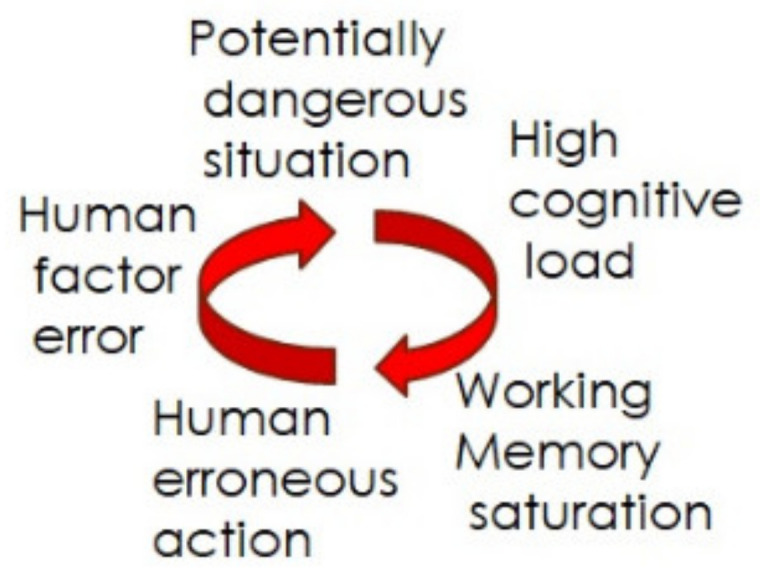
The high cognitive load results in human factor error and leads to the worst-case scenario.

A high workload, as per the ship’s collision reports, is induced by various disturbing factors, which typically distract the officer’s attention and response time in both urgent and non-urgent actions. The disturbance causes a high workload and working memory saturation, resulting in human-erroneous actions and human-factor errors (Figure 1), which brings us to potentially dangerous situations and, in one potential scenario, a collision [1]. The main mechanism of repeated simulation gain in terms of performance is based on the automatisation of behavioural processes and the progressive release of the working memory. With repetitions, some procedures become automated, decreasing cognitive load and thus releasing more working memory for other dimensions of the task; this is reflected in a higher level of performance. Thus, we simulated the typical pilots’ task and analysed the participants’ responses to the recurrence event.

In 2021, an accident occurred in the Suez Canal. The Egyptian authorities cited objective reasons (strong wind gusts) and implied “technical or human errors” [2] as well. Although there were no fatalities, the disaster led to enormous costs to world trade. The Suez incident was just one of many in recent years. According to the European Maritime Safety Agency’s (EMSA) annual marine casualty and incident survey, nearly 20,000 reports were recorded during the period 2014–2019; 28% had serious or even very serious consequences. General cargo ships were involved more than others, followed by passenger ships. A total of 41.5% of the cases were in port areas, where a pilot on board is compulsory. Most of the cases included human error as a primary cause [3]. 

Navigation in the port area is specific and typically accompanied by fatigue due to situational awareness of constraints such as channel width and depth, traffic in the port, weather, currents, etc., all of which add to cognitive load. The pilot, familiar with local sailing conditions, assists the ship’s captain with steering the ship safely to the berth. Since many studies rely on young and/or student participants, the question arises: are psychometric responses of inexperienced pilots (trainees) in real-time different from experienced ones? 

Studies show that stressful events affect the biometric responses of pilots. A study was conducted at Shanghai Maritime University on the effect of social cognition and risk tolerance on the safety behaviour of marine pilots. The results showed that awareness of the hazardous nature of the manoeuvre had a direct negative influence, while risk tolerance had an indirect negative influence on pilots’ safety behaviour [4]. Risk perception affects the pilot’s working memory saturation, which indirectly leads to human errors [5]. The saturation of officers’ working memory is caused by situational awareness, information recognition, priority setting, and decision-making processes during the task, e.g., navigation and collision avoidance. Additionally, a high workload is caused by various disturbing factors that divert an officer’s attention. Typical disturbing factors during navigation are bridge alarms and various calls and e-mails, which affect the navigator’s situational awareness [6,7,8]. The density of the disturbing factors (in each time window) increases the cognitive load.

Research into human factors in navigation and/or collision avoidance, including assessment of cognitive load during the task, is normally conducted in a full-mission simulator for objective reasons. The related work shows three typical methodological data collecting approaches: basic, direct, and indirect. The advantages and disadvantages of each approach will be explained in detail in the next section. After careful study of the available literature, we decided to choose the adaptive indirect methodological approach in order to reduce negative excitement, artefacts, and false readings due to wearable sensors.

### 1.1. The Basic Approach

The basic approach includes measuring the overall participant response to the decision-making time during the collision scenario, which was, in this case, run at the Maritime Institute in Korea [9]. A longer than usual decision time indicates a high cognitive load. The advantage of this approach is that the algorithm used is easily integrated with existing nautical instruments such as an automatic radar plotting aid (ARPA) and an electronic chart display information system (ECDIS) to evaluate officers’ response time. In addition to the response time, the studies using this approach typically involve the self-reported measure called the task load index (TLX), an instrument for assessing participants’ emotional states at specific moments during the experiment [10,11,12]. The TLX index is well-known but suitable only if the participant is self-aware of his/her personal emotional state.

### 1.2. The Direct Approach

The quantification of officers’ cognitive load and stress during the navigation task by observing their stress hormone cortisol and brainwave intensity provides us direct insight into participants’ efforts during the task. However, the disadvantage of the cortisol method is the time lag of the results. The peak level of the stress hormone is reached 40 min after the event. Thus, it is unlikely that the method can be effectively used on ships [13]. The sophisticated brainwave monitoring by electroencephalograph (EEG) faces a similar challenge. The method is invasive even with the latest helmet sensors, and highly sensitive to artefacts and false readings. Thus, interpretation of the results from the invasive sensors is challenging due to the negative effect of bio-excitation, caused by the measuring sensor itself. Such recordings in the post-processing phase typically involve neural network algorithms which, during the machine-learning period, eventually recognize the noise in the readings [14,15,16]. 

### 1.3. The Indirect Approach

Recognizing and prioritizing the important navigational information in the officers’ working memory, situation awareness, and making appropriate decisions may indirectly affect the participants’ body response, which is manifested in excessive heart rate (HR), blood volume pulse (BVP), electrodermal activity (EDA), and pupil diameter. Related work found behavioural performances to be possible causes of such effects, which will be the scope of our future research [17,18,19]. 

BVP is a measure of heart rate based on the volume of blood flowing through tissues in each capture area. The photoplethysmography (PPG) sensor detects changes by illuminating the body surface with an infrared light-emitting diode. The red light is absorbed by the haemoglobin of red blood cells and reflected by other tissues. The amount of light that returns to the PPG photodetector is proportional to the relative volume of blood in the tissue. The magnitude of the BVP is derived from the raw BVP signal and indicates the current relative blood flow. The units of measurement are sensor-dependent and have no additional physiological meaning. 

HR is derived from the raw BVP signal by measuring the distance between the two consecutive beats, i.e., the peaks of the raw BVP waveform. 

EDA refers to the electrical changes measured at the skin surface, which are influenced by signals from the brain. The EDA signal consists of two main components. The first component is the overall tone value EDA, which refers to the slower changing components and background features of the signal. The most common measure of this component is the skin conductance level (SCL), and changes in SCL are thought to reflect general changes in autonomic stimulation. The second component is the phase component, which relates to the rapidly changing signal element—the skin conductance response (SCR). The setting of the SCR detection threshold depends on the experimental conditions [20]. The mentioned parameters are typically used to determine arousal level and valence [21].

The studies proposing the indirect approach also involve eye tracking as a biometrical sensor, observing the number of eye fixations on a given object during the task, and the duration of fixation during the experiment. More fixations and longer fixation times are assumed to indicate a higher cognitive load. Disadvantages are related mostly to problems with the data collection, such as the specific and constant lighting condition requirements and the fact that participants must sit relatively still [22,23,24]. The studies using the indirect approach may additionally include wearable sensors and electrodes for bio-readings such as wristbands, chest electrodes, and finger sensors for monitoring the body response [25,26,27]. The participants’ none/low disturbance due to wearable sensors is an expected advantage of such an approach. A similar approach is used in [28], where stress and strain were measured on the container ships’ crew with the armband monitor and the biometrical watch. The conclusion was that the crew had a significantly lower heart rate during the sea passage than during the port stay or the river passage.

The paper is organized into five parts. After the introduction (Section 1), the sensors and methods are presented in Section 2. In Section 3, the results are presented, and in Section 4, the discussion. The conclusions is Section 5. 

## 2. Materials and Methods

To overcome the disadvantages of the previous approaches, we designed and implemented an adapted indirect approach. The key advantage of the adapted approach was to assess the marine pilots’ biometric response during the port approach simulation using non-invasive biometrical measures, such as the wristband multi-sensor. The main goal of this study was to analyse data from the pilots’ bio-sensor readings to determine how experience affects marine pilots’ biometrical response during the port approach. The primary observables, e.g., HR, EDA, and BVP, were post-processed and modelled. The advantage of using a non-disturbing biometric sensor was the decreasing noise in the recorded data, which made post-processing less complex and improved the accuracy of the results—especially useful as the data was collected outside laboratory-controlled conditions. Note that we do not model specific conditions from psych-physiological signals such as stress or cognitive load, but we compare psych-physiological signals directly. 

**Figure 2:**
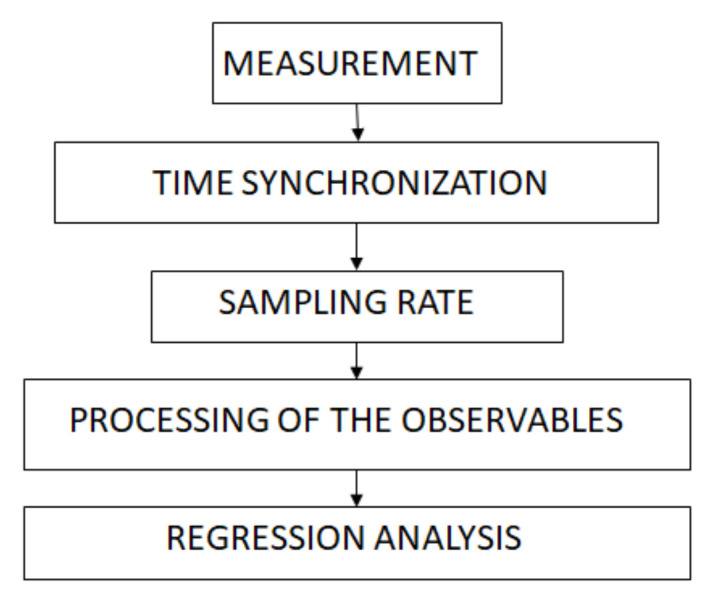
The data processing: synchronised data are resampled and processed before being statistically analysed.

We used the Empatica E4 multi-sensor wristband [20], a non-invasive and non-disruptive biometric data collection tool. In post-session interviews, participants typically mentioned that they did not notice they were wearing the sensor after the first few minutes, reinforcing the assumption that the disruptive effect of wearing a sensor is minimised. Data collection was initially wireless and in real-time. The wristband was connected via Bluetooth to a cell phone with a 5G Wi-Fi internet connection. The advantage of this configuration was real-time monitoring and data streaming. However, the disadvantage was that the participant could accidentally turn off the wristband and/or the cell phone, the phone could be updated during the session, the Bluetooth connection could be interrupted, the Wi-Fi connection could be interrupted, etc. Unfortunately, all this did happen, and we lost some valuable data. Therefore, we switched to a more robust design where we stored the measurement data locally in the wristband’s internal memory; after the simulation, the data was then transferred to the computer. Figure 2 shows basic data processing in the experimental design. The dataset consists of 7 Mb of BVP, 0.5 Mb of EDA, and 0.1 Mb of HR per session. The data streaming and visualization are in real-time, but post-processing is not. The experimental setup was not “user-in-the-loop” since there was no need to adapt the experimental setup in real-time. The estimation algorithm is of very low computational complexity and can be implemented in real-time on any device, including mobile ones.

### 2.1. The Sensor

We used three measures to assess biometrical data during the experiment. The first was a BVP sensor with a 64 Hz sampling rate. The strength of a blood vessel dilatation (vasodilation) is indicated by the signal amplitude, which is used as an indicator of the biometric response during task performance.

The second measure was the HR value, which was computed and excerpted from BVP inter-beat interval. The sampling rate of the HR was 1 Hz.

Finally, the wristband was additionally equipped with one pair of silver electrodes for monitoring EDA with sampling rate 4 Hz. In addition to BVP, HR, and EDA, the sensor also monitored the body temperature and wrist acceleration up to +/− 2 g with sampling rate of 32 Hz. The sensors were synchronized with a UTC time server.

The bridge simulator also had environmental sensors that collected data on humidity, temperature, and room noise. The environmental readings were made before and after each simulation to ensure equal conditions for all participants [29].

An ad-hoc questionnaire was used to collect participants’ personal information regarding gender, age, and prior navigation experience. 

### 2.2. The Design

The experiment was conducted in the Faculty of Maritime Studies and Transport, University of Ljubljana, Slovenia, where the Wärtsilä’s TechSim5000 simulator is installed, consisting of the modern and ergonomic full mission navigational main bridge together with four video projectors and two LCD monitors, providing a 270° of visual angle. Additionally, two simulated Voight-Schneider full-mission bridges (Tugs) assisted the large container ship during manoeuvring. The simulator environment was air-conditioned and ventilated for steady room temperature during the experiment. We found that important to avoid noise in the EDA and body temperature measurements, which are environmentally dependent. 

The participants differed by experience, which proportionally correlated with age (Table 1). They were divided into two groups, consisting of eight experienced male marine pilots with an average age of 47 years (SD = 6.8) and an average length of sea service of 11.9 years (SD = 4.1). In addition, four male trainees participated in the second group, with an average age of 25 years (SD = 1.6) and an average of less than one year of sea service.

The groups of participants, therefore, differed in both age and experience because we were not able to find young and experienced pilots or old and inexperienced pilots; the difference related to experience and age combined reflects real differences in terms of experience. Due to lockdown at the time of COVID-19, when the data were collected, the sample is small and unbalanced. We consider these shortcomings during stability analysis (saturated sample, bootstrapping statistics). Finally, the tugboat captains from Port of Koper volunteered to control and steer full mission tugboat simulators to increase the realism of the experiment. The tasks of the simulation were designed according to analyses of accident and traffic predictions in the northern Adriatic [30,31]. The simulations were run within six months and were repeated fifty times in total, which means that each participant repeated the simulation four times on average (Table 2). The average time between participants’ repetitions was 25 days. For post-processing, we selected only 21 port approaches by pilots and 11 by trainees due to inconsistent data recording and/or sensor issues.

The task of the participants was to take over the large container ship, bring her safely through the dredged channel into the port basin and, finally, to the designated berth. In practice, the marine pilots often face similar tasks when container ships approach the Port of Koper. The participants become acquainted with the simulated ship bridge and adapt to the environment during the initial learning phase. To increase the realism of the simulation, the task is as close to reality as possible. The beginning of the simulation (before any disturbing factor) is considered as control data for the analysis and comparison. Additionally, for observing the biometrical response over gained experiences, each session was used as control data for the next one. 

**Figure 3:**
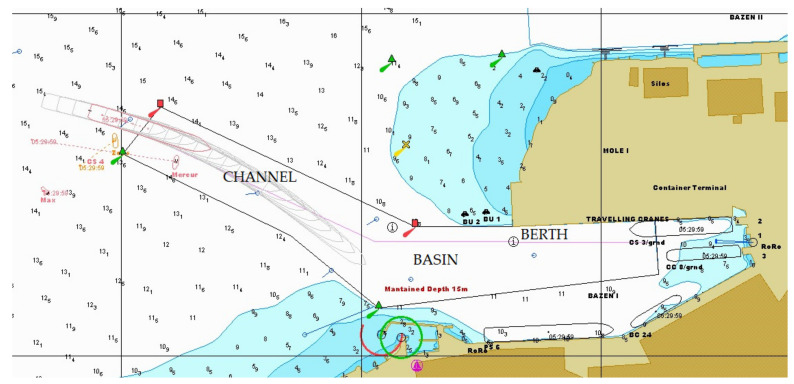
Port of Koper-approach to the container terminal. As seen on the electronic chart, the ship entered the canal at an undesirable angle due to strong wind. Two tugs assisted the 14 m draft container ship to stay on course within the dredged channel.

During the task, the participants encountered two types of disturbing factors. The expected disturbing factors were strong NE wind gusts during all phases of the task, pushing the large container ship off course, which would happen in Port of Koper regularly (one day per month on average). There were several unexpected disturbing factors, such as the tug accidentally cutting the towing rope, engine failure, etc. Staying on the desired course is the key to success due to the 14 m ships’ draft and the 15 m dredged channel depth, so there is not much room for error. As an example, Figure 3 shows a case of one participant’s late reaction to the wind gust, so the wind force pushed the large ship off course to the extent that she entered the channel at an unwanted angle. With the help of tugs and thrusters, however, the participants were able to keep the ship within the dredged channel.

The aim of the experiment was to monitor and record the pilot’s biometrical response throughout the task to gain insight into participants’ immediate reactions to the disturbing factors and provide an estimation of the participant’s response while performing the task. The special focus of the research was the biometrical response of two groups during the learning period (repetitions) with the aim of determining how experiences and learning periods affect body response. 

To provide the maximum verisimilitude possible, the pilot was giving orders to the ships’ helm and constantly communicated with both tugboat captains assisting the ship. The experienced senior instructor from the Maritime Administration represented the Port authorities, monitored the ships’ approach from the instructor console with an exclusive right to abort approach, and was giving orders to escape the manoeuvre for the sake of safety reasons, e.g., to prevent collision, grounding, pollution, or damage when weather conditions became too severe. The design ensured a high degree of realism. The task was finished when the ship was safely moored in the port, grounded, collided, or finished the escape manoeuvre. If all went well, the manoeuvring task typically took half an hour. After the task, debriefing followed, wherein the instructor and participant exchanged opinions and suggestions to absorb the experience for the next attempt. The aim was to determine the participants’ biometrical responses in the specific situation. The results are shown in the next section.

## 3. Results

Expectations of the quality of the results in this study are based on the assumption that professional participants are as engaged in the simulation as they are in real life. The biometric response of the pilots during the task was measured using the non-invasive wristband sensor. 

### 3.1. Time Setting

**Figure 4:**
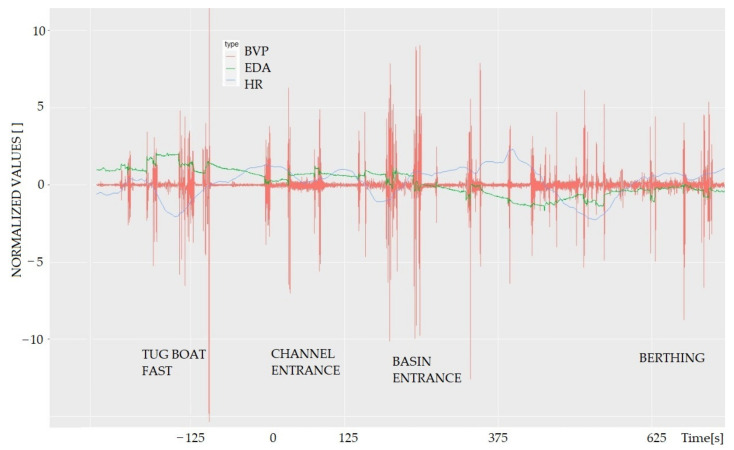
The normalized biometrical response of the experienced participant “1” during the port approaching task. The pattern is similar to all participants. The biometrical intensity typically correlates with situational awareness, given orders, and the ship’s position. Time “zero” is marked at the exact moment when the vessel crosses the line between the buoys at the channel entrance.

Synchronisation of the sensors was developed using a UTC time server, but the different sampling rates of the sensor recordings (64 Hz, 32 Hz, 4 Hz, and 1 Hz) required resampling to allow analyses at the same time axis (Figure 4). The scripts were written in the Python environment. The time “zero” was anchored at the exact moment when the ship’s recorded position crossed the line between the buoys at the entrance of the channel. The reason for this design is due to the different ships’ approaching speeds of each participant.

### 3.2. The Biometrical Response of the Experienced Pilots

The processed data from each session consist of BVP, EDA, and HR signals, as shown in Figure 3. The intensity of the observables during the port approach typically correlates with situational awareness, commands given, and the position of the vessel, e.g., entering the channel, entering the basin, and berthing, where the amplitude of the signals is highest. The normalization presented for each observed variable was created using the mean and standard deviation of the entire signal. 

### 3.3. The Effects of Pilot Experience on HR and EDA: A Case Study

For observing the effects of the gained experience, the visualization of the participants’ HR and BVP was conducted. The hypotheses were that the typical response and the excitement would lower over the sessions due to gained experiences, which we confirmed. However, we noticed that individual biometrical parameters could vary (e.g., the HR shows relatively average value due to gained experience, but the EDA is overexcited due to disturbance). Thus, a holistic approach is needed.

**Figure 5:**
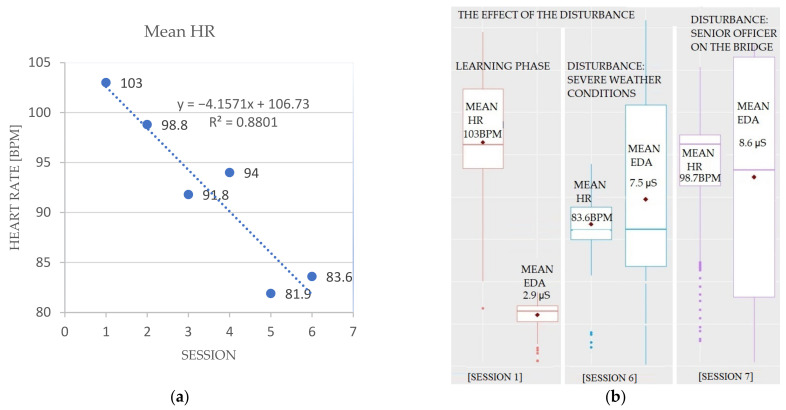
Observing the mean response of one of the experienced participants during seven simulations. The experience gained through the learning period reasoning decreased the HR (**a**); the EDA response correlates with the repetitions. The severe weather conditions in session 6 induced a higher EDA response. Session 7 shows an even higher response due to the instructor’s comments during the experiment (**b**).

Figure 5a shows the HR response of one of the participants during the learning phase (sessions 1 to 6). The data show that HR decreases during the first three sessions. Session N4 has an unusually high HR compared to the other sessions. The session, unlike the other sessions, was recorded in the afternoon, after the lunch break, which probably influenced the measured values [29]. In session N6, the pilot encountered a problem when the strong gusts of wind cut the mooring ropes during the mooring manoeuvre. Therefore, due to regulations and port safety reasons, the port authorities ordered the immediate “escape manoeuvre”. Despite this chaotic situation, the experienced pilot successfully completed the task. The measurements, however, also showed increased EDA (Figure 5b).

The increased response in session 7 (Figure 5b) was caused by an experienced instructor coming onto the bridge during the simulation and commenting on the pilots’ commands. As the data show, the instructor’s comments resulted in a biometric response from the pilot, both HR and EDA.

### 3.4. Pilots vs. Trainees

The last part of the Results section compares the average biometric responses between experienced pilots and trainees in the learning phase without unexpected disturbances as recorded during the first three sessions. In Table 3, the mean heart rate, blood volume pulse, and electrodermal activity are presented when the participants were in the inactive state (before the first navigation event started).

**Figure 6:**
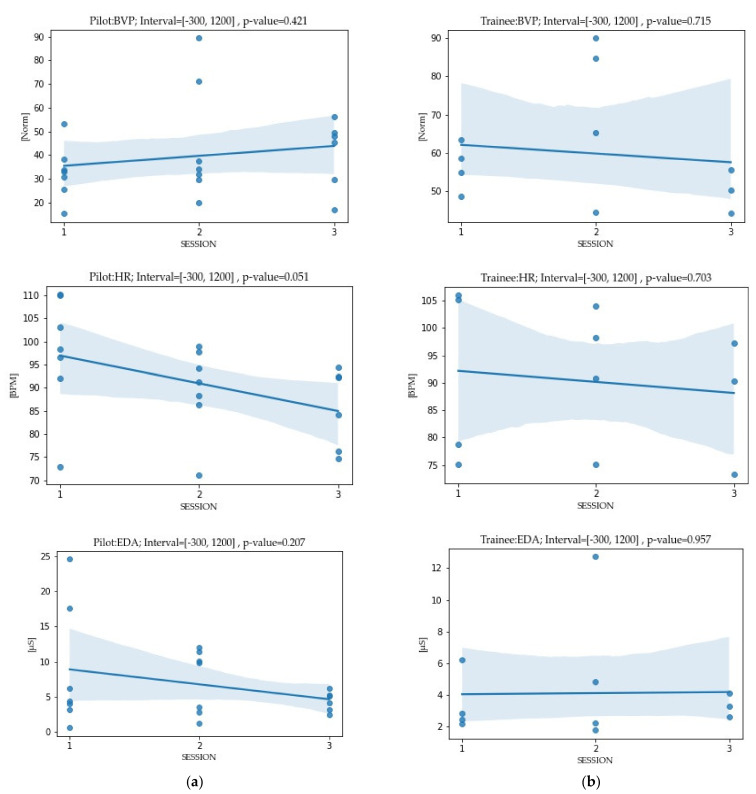
Mean biometrical response in the first three sessions of the experienced pilots; N = 8 (**a**) vs. trainees; N = 4 (**b**) without unexpected disturbances. The patterns of biometric response in the trainees with a lack of sea experience is different from that of the experienced ones. To control for individuals’ entering a biometrical state, the observing interval is chosen from −300 s to 1200 s of the first three sessions, where time zero represents the ship’s entrance into the channel. The *p*-value represents the regression line’s slope. Contrary to our expectations, the significant differences were not satisfactorily revealed.

Since the number of participants was low, we used nonparametric statistical tests Mann–Whitney U. A difference between eight experienced pilots and four trainees over the interval −300 s and 1200 s with respect to time of channel entrance is shown in Figure 6. As expected, the biometric patterns of the experienced participants (Figure 6a) had the highest values in the first session, which decreased over the repetitions. The decline changed by 0.2, 0.05, and 0.4 for EDA, HR, and BVP, respectively. As can be seen in Figure 6b, the trainees’ biometric response in the first experiment was relatively low compared to the pilots’ response. Within the first three repetitions, the *p*-value was high, so the data showed no significant change in the trainees’ biometric response (*p* > 0.005). We noticed that the approach did not satisfactorily reveal significant differences, and the *p* values lower than risk level α indicate a significant association since such low values can result from pure chance. For that reason, we applied a different approach.

### 3.5. The Significant Differences in the Biometrical Response

Because our data were not normally distributed, we applied the Mann–Whitney U statistical test to detect significant differences using the Python library Scipy ver. 1.7.3. Post-hoc (achieved) statistical power was assessed using GPower ver. 3.1.9.4. The anchored time-point was set to the first stressful test event at the moment of channel entrance, which was designated as time zero (cf. Figure 3). All measuring systems were normalized by subtracting the mean values of all signals measured from test participants when they were in the non-disturbing state a few minutes before the first stressful event. The non-disturbing state was determined by measuring the biometry of the participants at the beginning of the experiment, before the perturbing events. The estimated selected signal features were quantified to highlight the participants’ response to the experimental port-approaching interval. The interval started one minute before the anchoring event to typically twenty min after the event. The aim was to compare the BVP, EDA, and HR results of all pilots with the results of all trainees. According to the literature [32,33], we decided to extract the following features of the logged values: (a) mean value (mv); (b) standard deviation (std) by measuring the spread of the distribution for each signal of the sorted values compared to the mean; and (c) skewness (skw) by measuring the symmetry of the distribution, which indicates whether the values tend to gather around lower values (negative skewness), around the mean (zero skewness), or toward higher values (positive skewness). 

The results of extracted features are presented in Table 4. After three experiments, we obtained n1 = 21 pilot and n2 = 11 trainee measurements. We found that there were two significant differences between pilots and trainees. The standard deviation of the BVP signal was significantly lower in pilots (62.13) than in trainees 86.12), *p* = 0.002. Next was the significantly different skewness of the EDA signal for pilots (−0.87) compared to that of trainees (−1.78), *p* = 0.02. The results indicate significant differences in body response between the two groups. The reasons behind these differences are addressed in the discussion section.

Because the sample size is relatively small, we estimated the effect size (ES) of each measured difference between signal feature difference among trainees and pilots and also its post hoc statistical power (pw, meaning 1-pw is the probability that we did not detect the difference if it was present). We observe there are differences with relatively high effect size and low post-hoc power. This observation indicates that, in these cases, differences between trainees and pilots are likely there, but we did not detect them. Such differences are the mean of the EDA signal (es = 0.48, pw = 0.34), the mean of HR (es = 0.35, pw = 0.23), and the std of EDA (es = 0.82, pw = 0.68).

## 4. Discussion

The aim of the research was to gain as much verisimilitude involved in a port approach simulation as possible with insight into the participants’ biometrical responses regarding the experience gained through repetitions and the learning phase. Using simulators is a typical way to train pilots. Learning procedures normally include several repetitions. Although these learning repetitions on the simulators are an essential part of the learning process, there is very little literature related to the processes behind these repetitions. The aim of our study was to address, partly, an invisible dimension of pilots’ performance, the biometrical response, which is likely to indicate mechanisms behind decision-making in the berthing procedures. For this reason, we believe the paper contributes to our knowledge of how to make training sessions for pilots as effective as possible. The gathered results yielded two important findings. First, the participants’ biometrical response to the disturbance factor indicating correlation to unfavourable situations is measurable. Second, the trainees showed a different pattern of biometrical response on a simulator to that of experienced pilots; whereas the response in experienced pilots decreased with repetitions, the response in the trainees was relatively low and stable throughout the sessions. 

As mentioned, the trainees’ biometric response in the first session was relatively low compared to the pilots’ response, suggesting perhaps that the trainees were not as excited or focused as the experienced pilots (Figure 5). BVP mean values are reasonably low because the mean of the group is computed from the means of the signal of every individual, which was previously normalized to the mean value during the initial adaptation phase. However, the reason for the difference between experienced pilots and trainees is not clear. We suggest two possible explanations: (a) the pilots were aware of the severity of the task and the possible consequences and therefore took the simulation more seriously, while the trainees, not having real experience in situations that posed real challenges with real consequences, naturally were unable to block out the fact that the simulation is indeed a mere exercise; (b) because of the lack of experience, trainees are unfamiliar with the responsibility and danger, so the task does not trigger their biometric response. In any case, the results may have important implications for the future study of training procedures, which must consider the likelihood that novices do not deeply feel the real-life circumstances they are simulating. 

If trainees have significantly different body responses to different events on the bridge, they may not be included in studies simulating experienced pilots. To study pilots’ and marine officers’ responses during the repeated learning sessions, only experienced participants should be drafted; to study training procedures for the novices, only inexperienced participants should be studied. 

The study shows that the biometrical response decreases with the repetitions in the experienced pilots, suggesting that simulation with a high degree of realism reflects the biometric response of the pilots during the berthing procedures. The multi-sensor wristband [23] proved a useful tool for collecting biometrical data, yielding a profound insight into the participants’ bodily reactions in real-time. For this reason, we believe that non-invasive and non-disturbing multi-sensor tools such as the wristband have great potential for supporting officers on the bridge to efficiently cope with possible distress that may occur during difficult tasks and dangerous decision-making events on the bridge. 

The study also implies some challenges related to stress and cognitive load quantification, which would be interesting for future research. The first challenge refers to the pilots’ personal differences. Participants are not a homogeneous group in terms of intelligence, temperament, and stress-generated responses. Personal traits may function as a moderating factor between disturbance factor/stress events and pilots’ behavioural responses. Thus, to prove the assumption that personality is a moderator between stress events and pilot’s behaviour, the test of biometrical response of pilots under different stress-related conditions, controlling for their personality within the Big Five model of personality, must be performed [34]. 

The second challenge refers to time-series analyses that could be run from biometric data collected during the simulations of berthing procedures. In these analyses, biometric responses to the tasks within each session, which require high cognitive loads or are associated with high emotional arousal, could be compared to the biometrical response during the tasks with lower cognitive load or lower emotional arousal to test the dynamic aspects of decision making on the bridge. 

The third question relates to the technical challenges of multi-sensor data collections. The current study posed a number of challenges; we designed the study to monitor the data in real-time using a Wi-Fi connection but encountered several technical problems such as a temporal loss of internet connection and consequent loss of data, issues with sensor equipment such as the phone, which ran an update during the session, and in some cases, the wristband was switched off accidentally, thus causing loss of data, etc.; the experimenter’s immediate intervention may resolve the problem in terms of data collection, but interrupts the pilot’s problem-solving behaviour, brings in noise and thus affects the content validity of the data. In addition to that, synchronisation of all sensors with different sampling rates requires resampling and normalisation algorithms developed for each combination of sensors. Finally, the data from the multiple sensors with their temporal dimension, provide us with additional challenges. The current study provides several solutions to these problems that can be used for future interdisciplinary studies in sensor technology, marine studies, and psychology. The results can be provided as risk indicators for various analyses of navigation accidents where human error is the cause of an accident [35], or for other risk models where the human factor should be included or at least considered [36]. Furthermore, such a study is a valuable contribution to further studies on determining the appropriate speed of vessels in the port area, as pilots are an essential link in solving the problem of berth allocation or controlling large vessels by tugs and other stakeholders in manoeuvring. [37,38]. It is important to point out that virtually all studies are linked to real-world circumstances that may involve local authorities and industries. While writing this paper, we made several proposals to the port authorities, all of which have been accepted and are already being implemented. Currently, there are always two pilots on duty at the port, as we found that the pilots were overworked and clearly under excessive stress at times. Also, through our work and studies, we have found that better weather information is needed, which is now the case, and, very importantly, that the speeds in the ports were too high; now the speed limit is 6 knots when approaching the port and 5 knots inside the basins. As for manoeuvring with large ships, that is, very large container carriers, navigational devices (Independent Pilot Navigation Systems) are now available to pilots, and manoeuvres with these ships start a mile earlier, so pilots can prepare for entry, all of which reduces stress and increases safety.

## 5. Conclusions

The study reports the biometrical response of experienced pilots and inexperienced trainees during the port approach. The measures composed of HR, BVP, and EDA were used to measure the participants’ responses to the recurrence task. The scope of the research was to use as realistic a port approach simulation as possible to determine as much as possible a realistic biometrical response. The participants used a full mission simulator to navigate a large container ship and bring her through a dredged channel into a port basin to a designated berth. The meaningful finding is that trainees’ biometric responses in the simulation are different from those of the experienced pilots, so their results in the studies are not necessarily representative of professionals. The additional goal of this study remains to build a machine-learning (ML) algorithm to recognize the state of participants’ behavioural patterns in predicting decision-making processes and human-related errors in the berthing procedures. The health situation related to COVID-19 was persistently working against us. Thus, this part of the experiment is to be continued.

## Figures and Tables

**Table 1:** The participants’ detailed data: the experienced group’s average age is 47 years (SD = 6.8) with 11.9 years of sea service (SD = 4.1); the trainees’ average age is 25 years (SD = 1.6) and without significant length of sea service.

Pilot	Gender	Age	Sea Service [yrs]	Trainee	Gender	Age	Sea Service [yrs]
1	M	41	6	1	M	24	-
2	M	41	15	2	M	23	0.4
3	M	54	7	3	M	26	-
4	M	58	13	4	M	27	0.5
5	M	49	18	**Average**		**25**	**0.45**
6	M	37	8				
7	M	45	16				
8	M	51	12				
**Average**		**47**	**11.9**				

**Table 2:** Table of the participants.

Group	Number of the Participants	Number of Port Approaches
Marine Pilots	8	31
Trainees	4	19
Tugboat Captains	2	all 50

**Table 3:** Mean of features (mu and std) means by experimental groups of trainees and pilots when in an inactive state (before the first navigation event started).

	HR		EDA		BVP	
	Mean	std	Mean	std	Mean	std
**Trainees**	91.26	4.19	5.95	0.61	−0.05	57.01
**Pilots**	89.26	4.44	3.24	0.2	−0.01	87.8

**Table 4:** Statistically extracted features: the standard deviation of the BVP and skewness of the EDA is significantly different in pilots as opposed to trainees. The differences with relatively high effect size and low post-hoc power indicate undetected differences between the internal features of groups of pilots and trainees, mean value (mv), standard deviation (std), and skewness (skw); affecting the intermediate features—*p*-value (*p*), effect size (es), and post hoc statistical power (pw).

Feature f:	Mean Value (mv)				
	Pilots	Trainees			
**Signal**	**Mean**	**Mean**	***p***	**es**	**pw**
BVP	10.45	12.45	0.42	0.138	0.09
EDA	2.46	1.38	0.26	0.48	0.34
HR	0.21	2.48	0.08	0.35	0.23
**Feature f:**	**Standard Deviation (std)**				
	Pilots	Trainees			
	**mean**	**mean**	***p***	**es**	**pw**
BVP	62.13	86.12	0.02	0.82	0.68
EDA	1.77	0.87	0.23	0.82	0.68
HR	6.15	6.49	0.38	0.09	0.08
**Feature f:**	**Skewness (skw)**				
	Pilots	Trainees			
	**mean**	**mean**	***p***	**es**	**pw**
BVP	4.99	4.91	0.33	0.047	0.06
EDA	−0.87	−1.78	0.02	0.55	0.41
HR	0.22	0.04	0.17	0.22	0.14

## Data Availability

The study did not report any open source data.
